# Time course images of cellular injury and recovery in murine brain with high-resolution GRIN lens system

**DOI:** 10.1038/s41598-019-44174-7

**Published:** 2019-05-28

**Authors:** Chelsea D. Pernici, Benjamin S. Kemp, Teresa A. Murray

**Affiliations:** Center for Biomedical Engineering and Rehabilitation Sciences, Louisiana Tech University, Ruston, Louisiana USA

**Keywords:** Optical imaging, Preclinical research, Translational research, Regeneration and repair in the nervous system, Chronic inflammation

## Abstract

Time course, *in vivo* imaging of brain cells is crucial to fully understand the progression of secondary cellular damage and recovery in murine models of injury. We have combined high-resolution gradient index lens technology with a model of diffuse axonal injury in rodents to enable repeated visualization of fine features of individual cells in three-dimensional space over several weeks. For example, we recorded changes in morphology in the same axons in the external capsule numerous times over 30 to 60 days, before and after induced traumatic brain injury. We observed the expansion of secondary injury and limited recovery of individual axons in this subcortical white matter tract over time. In another application, changes in microglial activation state were visualized in the penumbra region of mice before and after ischemia induced by middle carotid artery occlusion. The ability to collect a series of high-resolution images of cellular features of the same cells pre- and post-injury enables a unique opportunity to study the progression of damage, spontaneous healing, and effects of therapeutics in mouse models of neurodegenerative disease and brain injury.

## Introduction

Multiphoton microscopy together with promoter-directed expression of fluorescent proteins for cell-type specific labeling has propelled *in vivo* neuroscience research into previously inaccessible regions of the animal brain. These enabling technologies have been combined with implantable gradient index (GRIN) lenses to extended the reach of multiphoton microscopes from the upper layers of the neocortex into the depths of subcortical structures of the murine brain^1–3^. GRIN lenses have been used to observe diverse phenomena, such as the growth of tumors^4^ and intracellular calcium dynamics^5^. Here, we present a system to use GRIN lenses for imaging progressive, secondary damage after brain injury, such as traumatic brain injury (TBI) and ischemic stroke, in murine models.

TBI has an immediate effect on brain tissue, including stretching and shearing injuries of vulnerable neuronal axons resulting in transport disruption or disconnection^6–8^. Shortly afterward, a cascade of cellular responses, called secondary injury, occurs that amplifies this initial damage manifold^9–11^. Axonal undulations are the first morphological sign of cellular damage. Later, swellings, called varicosities, develop on dendrites and axons. Further, damaged axons can disconnect, forming a larger swelling at the proximal end called a terminal bulb^6–8,12^.

Ischemic stroke is another brain injury that causes immediate damage, including cell death in the ischemic region. This is followed by inflammation-mediated secondary cellular damage in the surrounding tissue, called the penumbra^13^. A hallmark of this inflammation is activation of microglia, the resident immune cells of the central nervous system^14^. Morphologically, activated microglia transform from small cells with long ramified processes to cells having short processes with a brushy or ameboid form, which enables their mobility^15^.

Here, we present the design and use of an implanted small-diameter, high-resolution gradient index (hrGRIN) lens system for longitudinal studies that is optimized to image changes in fine cellular features associated with secondary injury in murine models of TBI and ischemic stroke. This chronically-implanted lens system, for use with multiphoton microscopy, has the temporal resolution to study dynamic events ranging from seconds to months. Using this system, we were able to observe mobile microglia in the penumbra region a day after ischemic stroke and the progression of secondary axonal damage over several weeks after TBI. We describe how to integrate this system with murine models of brain injury, including midline fluid percussion injury (mFPI), a model of diffuse brain injury^16^, and middle carotid artery occlusion, a model of ischemic stroke^17^. Pre-injury images, acquired in three dimensions (z-stack images), were used to establish an individual baseline for each mouse. Z-stack images were acquired periodically after injury to determine the time course of secondary cellular damage. Given the ability to monitor the same cells over multiple time points, this system will be useful for studying the effects of potential therapeutics on arresting neurodegeneration and promoting recovery.

## Results

### hrGRIN lens system for longitudinal, high-resolution images of deep brain regions in the same mouse

**Figure 1:**
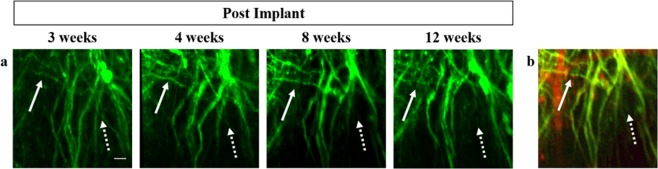
Longitudinal imaging using hrGRIN lens system. Chronically implanted hrGRIN lens assemblies can be used to image the same cellular features over the course of several weeks using multiphoton microscopy. (**a**) Images were acquired at 3 weeks post-implantation and then subsequently at 4 weeks, 8 weeks, and 12 weeks. Common features are identified across imaging sessions, such as two axons running parallel to one another (solid arrow) and a bundle of axons resembling crow’s feet (dashed arrow). (**b**) Texas Red dye conjugated to dextran was injected via the tail vein to demonstrate the ability to use injectable dyes as a fiduciary marker (red). Each image is a z-projection of five image planes, separated by 2 µm. Scale bar denotes 10 µm for all images.

We have exploited the inherent optical sectioning ability of multiphoton microscopy (MPM) and combined it with a modified GRIN lens and our TRIO imaging support system^18^ to enable visualization of the same set of cellular processes in three dimensions (3D) over repeated imaging sessions spanning a period of several weeks. We have used this procedure with transgenic mice expressing fluorescent protein in specific neuronal cells, such as pyramidal neurons, microglia, and astrocytes. Imaging was conducted at time intervals of one to four weeks over a period of up to 12 weeks. Originally, vascular counterstaining was used to provide landmarks to locate the same group of cells for each imaging session (Fig. 1b). However, we found that the patterns of neuronal processes were stable and as unique as fingerprints (Fig. 1a), obviating the need for vascular staining.

We extended the length of our singlet 0.5-mm diameter, 0.6NA GRIN lens^3,18,19^ to 2.6-mm in overall length by adding a glass window to the top of the lens. This provided the extra length needed to securely interface the lens with our TRIO imaging support system. The TRIO system provides secure head fixation to reduce motion artifacts from breathing, thermal support, and a nose cone for gas anesthesia, as described in Voziyanov *et al*.^18^. Furthermore, in previous work, we improved the axial resolution of this same GRIN lens by placement of glass, 0.2- to 1.0-thick, in the light path which enabled acute imaging of dendritic spines^3^. Additionally, using a glass window preserves the field of view of the singlet lens^3^ versus using a low-NA GRIN lens to extend the length^1^.

### Modification of hrGRIN lens system to collect longitudinal data before and after induced TBI

The novel method of combining the hrGRIN lens imaging system with the mFPI model allowed for the successful collection of both pre-injury and post-injury images. The mFPI model requires the installation of an injury hub. An injury hub is a Luer-lock connector that is affixed to the skull over a craniectomy to attach the fluid percussion device to the animal. It is removed after injury because it creates a portal for infection if the craniectomy is not sealed following injury. Additionally, the hub must be removed before *in vivo* imaging because it prevents positioning a microscope lens over the implanted GRIN lens.

**Figure 2:**
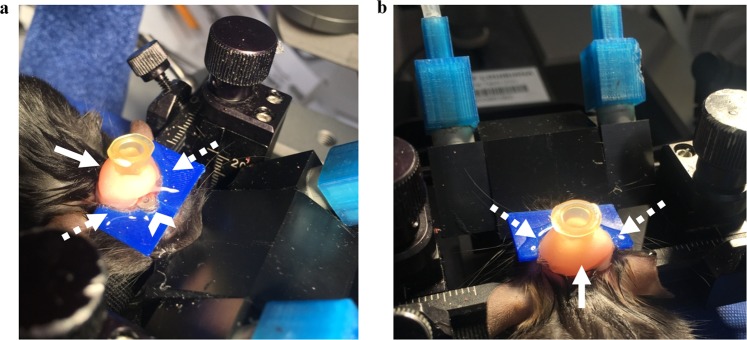
Installation of a fluid percussion injury hub with a custom head plate for the hrGRIN lens assembly. (**a**) An injury hub (Luer lock fitting) is attached over a craniectomy with a thin layer of rapidly curing elastomeric adhesive (Kwik-Sil, dashed arrows) to protect the GRIN lens (caret) and head plate (3D-printed plastic, blue) from unwanted detachment during the removal of the injury hub after injury. To prevent unwanted expansion of the elastomer during fluid percussion, a layer of rigid dental acrylic is applied over the elastomer taking care not to make contact with the head plate-lens assembly. (**b**) View of the back side of the injury hub showing the small amount of elastomer applied to the hub and head plate (dashed arrows) and the of dental cement (solid arrow) that is placed over the Kwik-Sil.

Traditionally, dental cement is used to attach an injury hub to the skull and then it is snapped off of the skull, en bloc, before the craniectomy is sealed^20^. However, the use of dental cement led to an unwanted extraction of the head plate (Fig. 2, blue rectangular device) and the attached GRIN lens assembly during the injury hub removal step. To prevent this, Kwik-Sil (World Precision Instruments, Inc.) was used to secure the injury hub to the 3D-printed head plate (Fig. 2a, dashed arrow). However, expansion of the elastomeric Kwik-Sil around the injury hub could lessen the fluid percussion force in an unpredictable manner. To prevent this potential expansion, a small amount of dental cement (Lang Dental Manufacturing Co., Inc.) was placed over the Kwik-Sil. Care was taken not to apply any dental cement to the 3D printed head plate (Fig. 2b, solid arrow). Dental cement prevented the Kwik-Sil from expanding during fluid percussion. Using Kwik-Sil as a base layer allowed for successful removal of the injury hub without perturbing the head plate and implanted hrGRIN lens assembly (Fig. 2a, caret).

### Collection of longitudinal images before and after induced TBI

**Figure 3:**
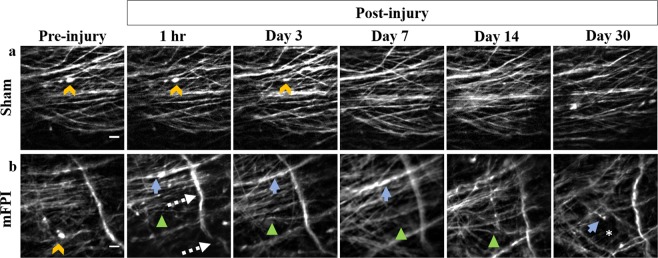
Time-lapse images following moderate midline fluid percussion or sham injury in Thy1-YFPH transgenic mice. hrGRIN lens assemblies were implanted just above the external capsule. Baseline images were acquired 1 week prior to induction of mFPI. Images of the same axons were acquired at 1 hr and at 3, 7, 14, and 30 days after mFPI. (**a**) Time-lapse images following sham injury. In sham animals, no apparent signs of damage were present in the series of images. The isolated swellings in the pre-injury images are normal and disappear over time (carets). These swellings are different from the multiple varicosities that appear on an injured axon after traumatic brain injury. (**b**) Moderately injured animals exhibited signs of axonal damage, including undulations (dashed arrows), disappearing axons (asterisk, 30 days), and axons with varicosities (solid arrows). Occasionally, undulated axons returned to baseline morphology and varicosities disappeared (arrowheads). Also, brighter fluorescent areas can occur where axons cross. Image z-stacks were examined to ensure that bright areas that look like varicosities were not axon crossings. Scale bars denote 10 µm for all images.

For two photon imaging before injury and through 30 days following mFPI, a set of equivalent imaging planes were collected at each of 6 time points, allowing comparison of identical axons over time for each mouse (Fig. 3). Indications of diffuse axonal injury were evident, including undulations (Fig. 3b, dashed arrows), varicosities (Fig. 3b, solid arrows), and disappearance of injured axons (Fig. 3b, asterisk)^7,21,22^. Pre-injury images were acquired one week preceding the induction of mFPI (Fig. 3a,b, pre-injury). These images provided baseline measurements of the number, positions, and shapes of axons. A few mice had one or two isolated swellings that disappeared over time (Fig. 3a,b, caret). These swellings are normal for adult rodents and are not associated with injury; they were excluded from post-injury measurements^23^. Another indication of injury is axon undulation. However, some axons naturally curve. Again, pre-injury images were used to exclude naturally curved axons from being counted as undulated axons after TBI.

**Figure 4:**
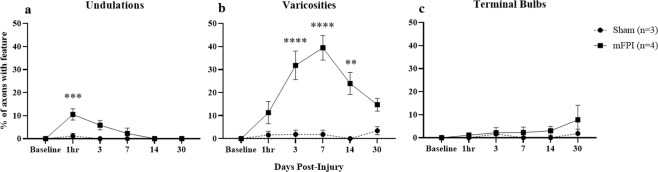
Quantification of axonal damage following mFPI. Common features (undulations, varicosities, and terminal bulbs) associated with diffuse axonal injury were evident following fluid percussion injury in mice. At each time-point the percentage of axons with each type of feature was quantified. (**a**) At 1 hr post-injury, mFPI mice had significantly more undulated axons than sham-injured animals (***p = 0.002). (**b**) At 3, 7 and 14 days post-injury, mFPI mice had a significantly higher percentage of axons with varicosities than sham-injured mice (****p < 0.0001, ****p < 0.0001, **p = 0.0012, respectively). (**c**) There was no statistical difference between sham and mFPI mice in the percentage of axons with terminal bulbs at any time-point. (Two-way ANOVA with Sidak correction.)

**Figure 5:**
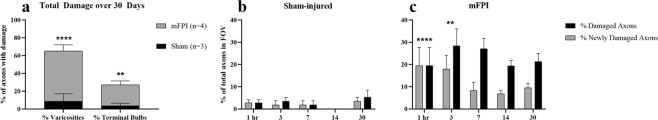
Progression of secondary damage. (**a**) Over the course of 30 days, mFPI mice had a significantly higher percentage of axons with varicosities (p < 0.0001) and axons developing terminal bulbs (p = 0.0017). (**b**,**c**). The percentage of newly damaged axons was compared, including new axons with varicosities, undulated axons progressing to axons with varicosities, axons with varicosities progressing to terminal bulbs, and terminal bulbs resulting in axon loss, that contributed to the total percentage of damaged axons at each time-point. As expected, the mFPI mice had a significantly higher percentage of axons in the field of view that were newly damaged (p < 0.0001 and p = 0.004, respectively) at the 1-hr and 3-days post-injury time points compared to sham-injured mice. Isolated swellings occasionally and transiently appear in the adult rodent brain, which likely explains their appearance in sham animals^23^. Two-way ANOVA with Sidak correction. ****p < 0.0001, **p < 0.01 vs. sham-injured.

In images acquired 1 hr after injury, mFPI mice had a significantly higher percentage of undulated axons as compared to sham-injured animals (p = 0.002, Fig. 4a). Undulated axons are identified as having a rippled profile as compared to their baseline morphology^21,24,25^ (Fig. 3b, dashed arrows). At 3, 7, and 14 days post-injury, mFPI mice had a significantly higher percentage of axons with varicosities as compared to sham-injured mice (p < 0.0001, p < 0.0001, p = 0.0012, respectively, Fig. 4b). Varicosities are identified as swellings along an axon fiber^6,25,26^ (Fig. 3b, solid arrows). The individual swellings are often connected to one another by a thin axon segment^25^. At each time point, there was no statistical difference in the percentage of axons that developed terminal bulbs between sham and mFPI mice (Fig. 4c). However, when comparing total terminal bulb development over 30 days, injured mice had a significantly higher percentage of axons with this feature (Fig. 5a, p = 0.0017).

To demonstrate the unique capability of our system to evaluate progressive secondary damage over time following injury, the percentage of axons with new damage was calculated at each of the five time points (Fig. 5b,c). By comparing successive images, we quantified the progression of secondary damage as the percentage of axons with new damage (i.e., progressing from an undulation to varicosities, or from varicosities to a terminal bulb, Fig. 5b,c). Injured mice had a significantly higher percentage of axons with new damage than the sham-injured mice at 1 hr (p < 0.0001) and at 3 days post-injury (p = 0.004), respectively. Some axons may have had damage outside of the field of view; thus, we occasionally observed the appearance of an injury feature, such as a terminal bulb that was not preceded by development of varicosities and axon loss not preceded by a terminal bulb. We also observed the straightening of undulations and the disappearance of varicosities (Fig. 3b, arrowhead) on a smaller percentage of injured axons over time, 12.5 ± 12.5% and 49.8 ± 9.0%, respectively.

Axon density and orientation varies naturally in the external capsule. This range is depicted in the different axon densities shown in Fig. 3a,b. An image set with fewer axons and a relatively large, dark background was selected for Fig. 3b because it is easier to see varicosities against a dark background. Despite the variability, we saw no difference in the total percentage of damage based on axon density (dense ≥25 axons in the field of view, n = 3, non-dense <25 axons in the field of view, n = 4) at each timepoint (p = 0.83 at 1 hr, p = 0.42 at Day 3, p = 0.3 at Day 7, p = 0.2 at Day 14, and p = 0.2 at Day 30, 2-way ANOVA with Sidak correction). This would be expected with a model of DAI^8,26^. This lack of a difference is also expected because the percussion site was in a caudal position and the lens implant site was in a lateral and rostral position.

In our hrGRIN lens assembly, the lens is affixed to the head plate to ensure optimal lens alignment when the mouse is placed in the TRIO platform for imaging. While this is an advantage for obtaining consistent, high-resolution images over time, it is a disadvantage if the head plate detaches. Over time, some head plates became detached after adhesive loosened. Only 1 of 8 mice in the TBI study lost its head plate before the 30-day time point. By 60 days after injury, which is 12 weeks after implantation, 3 mice had intact head plates and were imaged. For future procedures requiring end points beyond 30 days, we will use bone screws, placed laterally, as anchors to hold the adhesive more securely to the skull.

### Utility of hrGRIN lens system for stroke models and other dynamic processes

**Figure 6:**
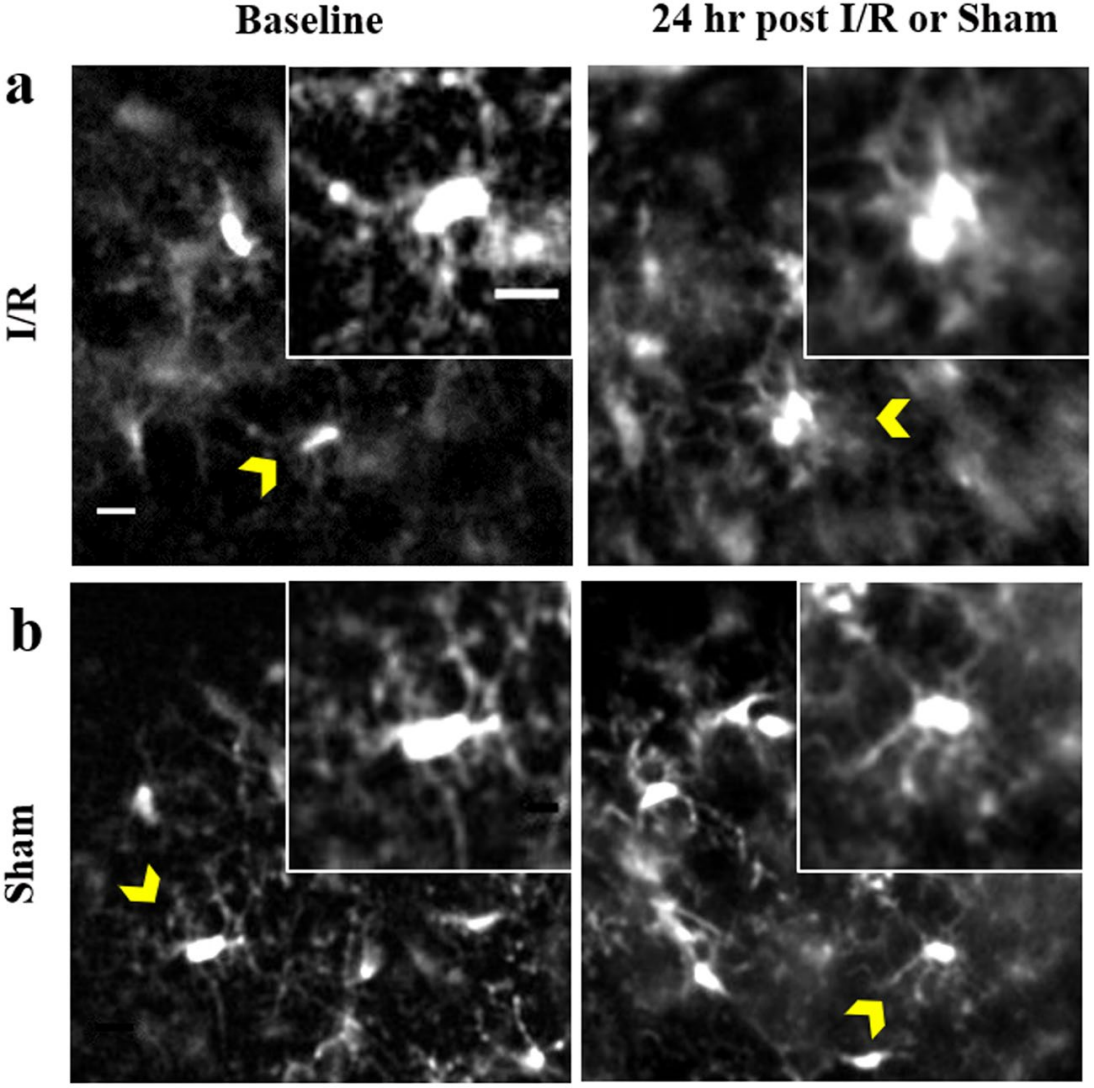
Representative images at baseline and 24 hours after sham or transient (60 minute) MCAo procedure. Microglia in the resting state have ramified processes, as seen in both baseline conditions (Baseline **a**,**b**) and in the sham injured mouse (Sham, **b**). When microglia become activated, their processes retract and the cells often exhibit a brushy or ameboid morphology (24 hr post-I/R, **a**). Insets are enlarged, representative images of microglia (caret) morphology. Images are z-projections of 5 × 1 µm optical slices. Scale bar denotes 10 µm, inset scale bar denotes 5 µm.

This imaging system is not limited to TBI studies; we have used in it variety of applications in our lab utilizing transgenic mice and injectable fluorescent dyes^18,19^. The system can be shortened to obtain images from the cortex in mice by omission of the window and direct attachment of the GRIN lens to a glass cover slip. For instance, microglia become activated and mediate secondary damage in the penumbra region surrounding the ischemic core after stroke^13^. To determine if our system could be used to detect morphological features of activation after experimental ischemic stroke, we used the filament model of middle cerebral artery occlusion (MCAo)^17^ in Cx3cr1-tdTomato mice that express red fluorescent protein in microglia. Three weeks after implantation, baseline images were acquired and then mice were imaged 24 hours after 60-minutes of ischemia followed by reperfusion (I/R). Baseline images showed microglia with long ramified processes characteristic of the resting state (Fig. 6a,b, baseline). After I/R, we observed microglia with brushy processes and ameboid morphology that is indicative of the activated state (Fig. 6a, 24 hrs post-I/R). In contrast, sham-injured mice did not have any features of activated microglia (Fig. 6b). In addition, cellular dynamics, such as activated microglia moving through tissue and monocytes and neutrophils flowing through a blood vessel can be recorded after MCAo (Video S1). These results demonstrate the utility of the hrGRIN system for use in murine models of inflammation and for evaluation of drugs to treat inflammation-mediated secondary damage after ischemic stroke.

**Figure 7:**
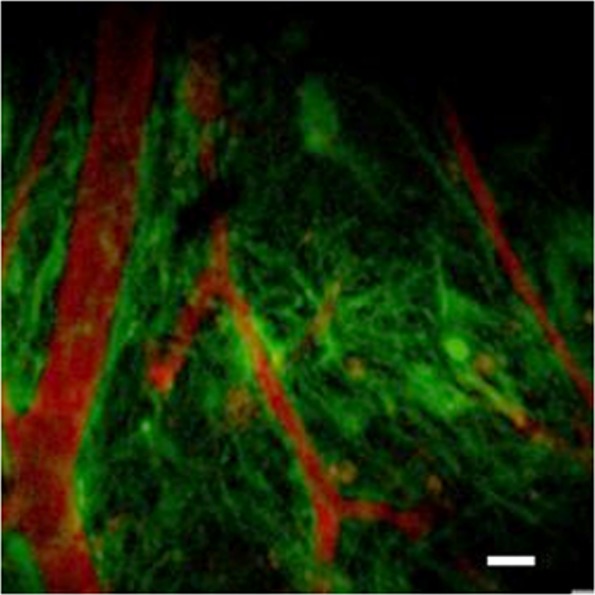
Multiple uses of the hrGRIN lens system. This system can be assembled without the additional glass window to image cortical layers 5–6. This modified version was implanted into GFAP-GFP (green) mice, and used with a vasculature counterstain, Texas red (red) conjugated to dextran. The system can be used in studies where astrogliosis or neovascularization would be of interest. Scale bar denotes 10 µm.

Following brain injury, reactive astrogliosis and vasculature remodeling can occur over time^27–30^. Using the shorter version of the hrGRIN lens, cortical astrocytes and the vasculature, stained with Texas Red conjugated to 70 kDa dextran (ThermoFisher Scientific Inc.), were observed in GFAP-GFP mice (Fig. 7). Stained vessels provide fiduciary markers to locate the same astrocytes over several imaging sessions, or a means to measure changes to the vasculature. This system would be useful in studies where the development and/or resolution of astrogliosis and neovascularization following injury are of particular interest.

## Discussion

The hrGRIN system for longitudinal imaging of deep brain regions was adapted for the fluid percussion model of diffuse TBI and for the MCAo model of stroke in mice. Each required a slight modification to the system. For the TBI model, this system has the spatial resolution to observe morphological indicators of primary and secondary axon damage^6,8,21^ and the temporal stability to enable imaging of the same axons over several weeks. Likewise, the system had the spatial resolution to observe fine microglial processes in a model of ischemic stroke and the temporal resolution necessary to observe motile microglial cells. Furthermore, baseline data acquired prior to induction of TBI was used to normalize data acquired after injury. This reduced variability and thus permitted the use of fewer animals in our study^19^.

High-resolution *in vivo* images acquired over multiple time points using implanted GRIN lenses fill a critically important spatio-temporal niche because the time course of important physiological changes after brain damage varies from seconds to weeks and longer^31–40^. Other imaging tools exist to observe damage at different spatiotemporal resolutions. For example, diffusion tensor imaging (DTI) reveals increased diffusion of water through damaged axons on the level of white matter tracts^35,41^, and histology identifies molecular biomarkers of damage and inflammation in cells^34,42,43^. However, DTI lacks cellular resolution and has low temporal resolution, whereas histology has the potential for exceptional subcellular resolution but lacks observation of dynamic processes. The hrGRIN lens system, presented here, augments these modalities by filling this spatiotemporal gap, to enable longitudinal imaging with resolution of fine cellular processes needed to identify cellular damage and to distinguish between activated and resting microglia.

Other high-resolution, optical techniques exist to collect longitudinal images in the mouse brain with two-photon microscopy, such as cranial and thinned-skull windows^44–48^. However, these techniques are limited to imaging the upper regions of the cortex due to light scattering in tissue. Three-photon microscopy has increased multiphoton imaging depth of cell bodies and some cellular processes to about 1-mm without the need for micro-optical implants^49^. However, resolution of cellular processes degrades with depth. To overcome the slow volumetric scan speed of three-photon imaging, axially-elongated Bessel-beam scanning has been used to achieve 5-Hz scan speeds in the mouse cortex (620–685 μm below the dura)^50^. Additionally, miniature, head-mounted microscopes incorporating implanted GRIN lenses have the capability to image intracellular calcium dynamics, at 10’s of frames per second. In contrast, multiphoton microscopy uses x-y scans which limits full-frame acquisition to only a few frames per second^5,51,52^. On the other hand, head-mounted microscope systems use shorter wavelength, single-photon excitation, which limits the depth of tissue that can be imaged versus multiphoton excitation^5,51,53,54^. Another *in vivo* imaging approach is to use thinner, multimode fibers combined with adaptive optics. Their more slender geometry provides a less invasive means versus GRIN lenses to reach deep brain regions for high-resolution, 3D imaging^55,56^. These systems have facilitated acute imaging as deep as the mouse thalamus with comparable resolution to a GRIN lens^55^. However, their small diameter (50–100 μm) results in much smaller fields of view than the hrGRIN lens, which is ~240 μm^3,55,56^.

GRIN lens implants can consist of a single lens, or they can be composed of one or two short, high numerical aperture (NA) focusing lenses combined with a lower NA relay lens for deeper imaging; these are referred to as singlet and compound lenses, respectively. For a range of lengths and diameters of compound and singlet lenses, the reader is referred to a review by Meng *et al*.^57^. Compound lenses combine a shorter, high-NA focusing lens (e.g., 0.8 NA) with a lower NA relay lens (e.g., 0.48 NA)^58^. This provides better resolution of fine cellular processes than typical 0.5-NA and 0.6-NA lenses. However, adding a lower NA relay lens to a high-NA lens reduces the field of view^1,3^. When comparing lenses with the same outer diameter, singlet GRIN lenses provide a three-fold or larger field of view than these compound lenses^3^. To maintain the maximum field of view of our 0.6-NA singlet lens, we used a glass window instead of a GRIN relay lens to extend the length of the lens. In our previous work, we placed a glass window in the laser beam path to compensate for spherical aberration which improved axial resolution over two-fold^3^. For the hrGRIN lens assembly, we directly attached a glass window to the GRIN lens instead of using a separate piece of glass. This provided the extra length needed to securely interface with our TRIO imaging support system, preserved the relatively large field of view, and enabled observation of fine cellular features such as varicosities.

An inherent limitation of imaging through GRIN lenses is that the field of view is restricted to a relatively small, fixed imaging field. Thus, it is important to place the lens in a region where cells will be widely impacted by the injury or neurodegenerative condition being studied. In the case of stroke and TBI, these regions are well known^13,42,59^. Also, inter-animal variability, which can be a problem in any study, could be especially problematic when examining a small brain region through a GRIN lens. We overcame this limitation to successfully discriminate between mFPI-injured and sham-injured mice. Using preinjury images, we counted the number of axons in the field of view and any preexisting features that could be confused with injury, such as curved axons and isolated swellings. After that, we accounted for the axons that were damaged, as a percentage of the total number of cells. This mitigated the effect of having different numbers of axons in a field of view for different mice. Our conclusion that peak damage occurred at 7 days agrees with other studies using histological approaches^6,59^.

After the initial inflammation from implantation of GRIN lenses subsides, a glial scar remains. Previously, we have shown that three weeks after implantation there is a 46-μm thick glial scar immediately under the GRIN lenses that contained some reactive astrocytes (positive for glial fibrillary acidic acid protein, GFAP+) and a few microglia with activated morphology that were positive for Iba-1^19^. This is consistent with another published study that showed a similar, 25- to 40-μm thick layer of GFAP+ cells under GRIN lenses^4^, Supplemental Methods. Notably, the imaging region of our lenses is 125 μm from the end of the lens and beyond; thus, imaged tissue does not include the glial scar^19^. Furthermore, images of sham-injured mice acquired over multiple time points for the present study show no signs of damage, supporting our earlier study that the lens does not promote inflammation in the imaged region. A few commercially-available lenses have a working distance of 50 μm; thus, inflammation could pose a problem. Another potential concern regarding inflammation is that the lens might exacerbate inflammation caused by experimental brain injury. We believe that there was relatively little or no additional inflammation induced by mFPI due to the distance between the injury site and the lens. GRIN lenses were implanted rostrally and laterally, and the FPI force was applied medially and downward in the caudal region of the brain. If injury had occurred due to forces against the lens, this would have resulted in a focal injury with more undulations and varicosities in the 1-hour images than were observed^25^. Furthermore, injured mice exhibited diffuse damage that peaked at 7 days after injury, as expected^26^. However, placing a lens next to the FPI site could result in shearing forces against the lens, which could exacerbate inflammation. In this case, analyses should be conducted beforehand to assess the level of inflammation to ensure that it is low enough to permit discrimination between treatment conditions.

The hrGRIN lens system with its 3D-printed head plate and TRIO imaging support system^18^ can be modified using a 3D design program to enable its integration with other research modalities, including neurochemical sensors^60^, microdialysis probes, and implanted electrodes. Future studies using siRNA, conditional knock-out mouse models, optogenetics, and genetically-encoded calcium indicators can be used to further elucidate the timing of molecular mechanisms of secondary injury and endogenous mechanisms of recovery. Furthermore, this system’s ability to directly observe movement and changes in cells over time, together with these other modalities, will enable a more nuanced understanding of the sequelae of neurodegeneration and regeneration. This longitudinal imaging approach will also enable identification of the effects of therapeutics over time and of transition points in injury or disease states for identification of treatment windows. With this versatility, and its high spatial and temporal resolution, the hrGRIN lens system can become a widely utilized platform for basic and preclinical research.

## Methods

The use of the hrGRIN system requires fabrication and assembly of 3D printed components, a standard multiphoton microscope, and stereotaxic surgery.

### Animal care and use

Male and female 8- to 9-week old mice were used for lens implantation. Thy1-YFHP mice were used for midline fluid percussion injury (mFPI) experiments. Cx3cr1-ROSA26-tdTomato mice were used for middle carotid artery occlusion (MCAo) and GFAP-GFP mice were used to visualize astrocytes. All experimental procedures with mice were performed in accordance with a protocol approved by the Louisiana Tech University Institutional Animal Care and Use Committee and in accordance with the National Institutes of Health Guide for the Care and Use of Laboratory Animals (NIH Publications No. 80–23, revised 1996). Animals were housed in a 12 h on - 12 h off light cycle. Food and water were provided *ad libitum*.

Mice used in this study were, as follows:

#### Thy1-YFPH mice

Breeding pairs for Thy1-YFHP mice were procured from The Jackson Laboratory, USA, stock number 003782, strain name B6.Cg-Tg(Thy1-YFP)HJrs/J^61^. Male mice, hemizygous for Tg(Thy1-YFP)HJrs were bred with non-carrier female of the same strain.

#### *Cx3cr1-ROSA26*-*tdTomato mice*

Cx3cr1 mice were obtained from the Mutant Mouse Regional Resource Center – UC Davis, USA (MMRRC_036395-UCD STOCK Tg(Cx3cr1-cre)MW126Gsat_Mmucd))^62^ and crossbred with ROSA26-tdTomato mice (Jackson Laboratory, USA, stock number: 007909 strain name B6.CgGt(ROSA)26Sor^tm9(CAGtdTomato)Hze^/J)^63^.

#### GFAP-GFP mice

Mice were supplied by The Jackson Laboratory, USA (stock number 003257, FVB/N-Tg(GFAPGFP)14Mes/J Pair)^64^. Hemizygous carriers were bred with non-carriers of the same strain.

### Assembly of hrGRIN lens

**Figure 8:**
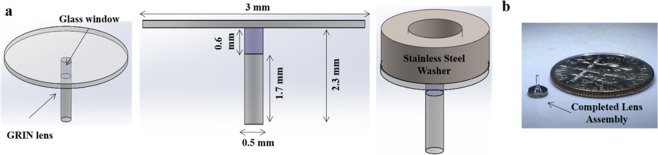
hrGRIN lens assembly. (**a**) SolidWorks schematics of hrGRIN lens assemblies. GRIN lenses (l = 1.7 mm, d = 500 µm) are affixed to a glass window (l = 600 µm, d = 500 µm) and #1.5 cover glass (d = 3 mm) with NOA 71 optical adhesive. A 1-mm thick stainless-steel washer (ID = 1.7 mm, OD = 3 mm) was permanently affixed to the cover glass. The stainless-steel washer portion of the assembly facilitated loading of the assembly into a cannula holder for stereotaxic implantation. (**b**) Completed assembly next to a small coin (US dime) for size comparison.

Each lens implant consisted of a 500-μm diameter, 1.7 mm long, 0.6 NA, ILH, uncoated GRIN lens, just under ½ pitch (Go!Foton, Somerset, New Jersey, USA). The lens was either affixed to a #1.5 glass 3-mm diameter cover slip (Electron Microscopy Sciences, Hatfield, Pennsylvania, USA), or it was attached to a 500-µm diameter, 0.6-mm long glass window^65^ (Bern Optics, USA) and then the window was affixed a 3-mm diameter cover slip. NOA 71 optical adhesive (Norland Products, Inc., Cranbury, New Jersey, USA) was used to adhere glass components^3,19^. The cover slip facilitates lens handling, seals the craniectomy, and corrects for some of the spherical aberration inherent in GRIN lenses^3^. The same adhesive was used to permanently affix a small, stainless steel washer, 1 mm thick to the cover slip (ID = 1.7 mm, OD = 3 mm, McMaster-Carr, Santa Fe Springs, CA) (Fig. 8a). Assemblies were cured with a UV lamp (160 W, UVB/UVA Self-Ballasted Mercury Vapor Lamp, Reptispa) for 90 minutes, with a 180° rotation at 45 minutes to ensure even curing of adhesive. The following day, additional adhesive was placed around the interface of the lens and cover slip (to protect against accidental separation) and cured again for 90 minutes, with rotation at 45 minutes. Completed lens assemblies (Fig. 8b) were stored in dust free boxes with desiccant.

### hrGRIN lens system implantation

**Figure 9:**
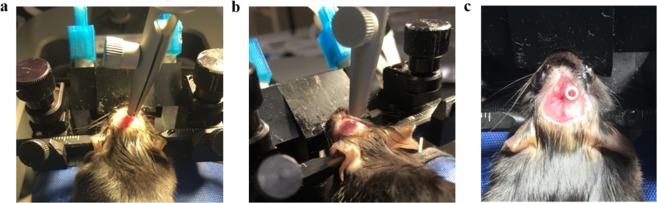
hrGRIN lens system implantation. (**a**) An assembled hrGRIN microlens is loaded into a cannula holder and stereotaxically aligned for implantation. (**b**) The top of the cannula holder is angled 15° away from the midline to ensure the system lays flat on the skull and then it is slowly lowered at a rate of 0.1 mm/min to minimize damage from implantation. (**c**) RelyX Aplicap (3M) dental adhesive is applied under the coverslip and around the skull after implantation. Once the adhesive has cured (~20 minutes), the cannula holder is removed.

Mice were briefly anesthetized with 5% isoflurane using a SomnoSuite® system (Kent Scientific Corp., Torrington, Connecticut, USA) and subsequently given an intraperitoneal injection of ketamine/xylazine cocktail (10 mg/kg ketamine and 1 mg/kg xylazine in 0.9% sterile saline). Mice were transferred to a stereotaxic frame (Stoelting Co., Wood Dale, Illinois, USA), equipped with ear bars and a nose cone for isoflurane delivery (Leica Biosystems. Inc., Buffalo Grove, Illinois, USA). To ensure the animal was fully anesthetized, noxious stimuli was applied every 15 minutes. Aseptic surgical techniques were used to make a midline incision, and a surgical bone scraper was used to remove the periosteum. A micro drill and 0.5 mm dental burr were used to make a 1 mm craniectomy over the right hemisphere of the cerebral cortex and the tip of a 28 G hypodermic needle was used, positioned at a low angle to the dura, to remove the dura mater^18,19^. The washer on top of the lens assembly was held by a cannula holder (David Kopf Instruments, USA) for stereotaxic insertion of the lens. The top of the cannula holder was angled 15° away from the midline and the lens was implanted 0.9 mm rostral to bregma, 2.25 mm lateral to the midline, and lowered ventrally 1.5 mm at a rate of 0.1 mm/min so that it was implanted just above the external capsule (Fig. 9a,b). The implant was secured to the skull with dental adhesive (RelyX Aplicap, 3M Corp) which was allowed to cure for 15 minutes before disconnecting the cannula insertion tool. This procedure immobilized the implanted lens assembly and the cover slip sealed the craniectomy (Fig. 9c). Mice were returned to their cages with a warm heating pad (Braintree Scientific, Inc., Braintree, Massachusetts, USA) and were monitored until they maintained sternal recumbency. To minimize post-surgical pain, an analgesic was dissolved in the animal’s water (Children’s Ibuprophen, 30 mg/kg/day in 8 oz of water) for 72 hours following the procedure. Mice were allowed to recover at least three weeks before imaging^19^.

### Head plate design and attachment

**Figure 10:**
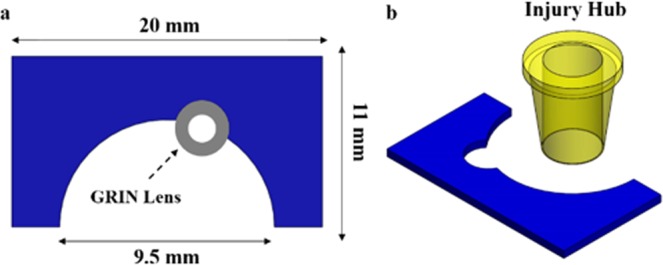
In house 3D printed head plate designed in SolidWorks® and compatible with the TRIO platform for imaging (**a**). Top view of the head plate with two cut-outs. A small cut-out (d = 3 mm, dashed arrow) at the front of the head plate allows the head plate to be easily aligned with the washer portion of the hrGRIN lens system. The large cutout (d = 9.5 mm) in the back allows access to the skull so that an injury hub (**b**) can be installed for fluid percussion injury.

Head plates were designed in SolidWorks® software and 3D printed in house with a MakerBot® Replicator 2® 3D printer. (See the 3D Print Methods and Data Sharing section for link to download the 3D print file). Head plates were batch printed with PLA filament (G-Star Technologies) with a layer height of 0.1 mm and 100% infill. The head plate was oriented flat, with its widest face touching the bed of the printer. This orientation allowed for optimal printing. The head plate is 20 mm × 11 mm × 1 mm (L × W × H) and has a cut-out (d = 3 mm) to fit over the washer affixed to the hrGRIN lens assembly (Fig. 10a). The back end (d = 9.5 mm) of the head plate is open to allow for the placement of an injury hub, a vital part of the fluid percussion injury system. The hub is attached during a subsequent surgery (Fig. 10b).

### Longitudinal imaging

A mouse was briefly anesthetized with 5% isoflurane using a SomnoSuite^TM^ anesthesia system (Kent Scientific Corp., USA) and then secured in a TRIO platform imaging support system, as previously described^18^. Prior to imaging, the cover slip on top of the implanted GRIN lenses was cleaned with the aid of a stereo microscope using 91% isopropyl alcohol and a small wooden stick, such as the blunt wooden end of a cotton-tipped applicator. Anesthesia was maintained for the duration of the imaging session with 1–1.5% isoflurane and air. The mouse, secured in the TRIO platform, was moved to the stage of a multiphoton microscope system. The head plate was positioned under a microscope objective using brightfield mode and 10X eyepieces to center the implanted GRIN lens by manually moving the TRIO platform. After this, the microscope objective was moved up and down in the z plane using epifluorescence mode and a green emission filter to locate the top of the implanted GRIN lens. The z position of the objective lens was then raised 150 μm which is the optimal focal distance of the GRIN lens; this position was recorded.

A Chameleon Vision-2 multiphoton laser (80 MHz, Coherent) tuned to 890 nm was used to excite YFP (yellow fluorescent protein), GFP (green fluorescent protein), FITC (fluorescein isothiocyanate), and Texas Red fluorescence. The laser was tuned to 1020 nm to excite tdTomato fluorescence. Emitted light was filtered through a green fluorescent protein filter (FF02-525/40 Brightline^®^ filters, Semrock, Inc.) for YFP, GFP, and FITC. Light was filtered through a red fluorescent protein filter (FF01-612/69 Brightline ®, Semrock, Inc.) for tdTomato and Texas Red. Laser power ranged between 9 and 24 mW at the sample, which is within accepted limits to avoid photodamage. The lowest laser power was used for the planes closest to the end of the lens and power was increased for imaging planes that were deeper into the tissue^4,58^. Scanned images were acquired on a Vivo-2 upright, multiphoton microscope system equipped with GaAsP detectors (Intelligent Imaging Innovations, Inc., Denver, Colorado, USA), a Pockels cell (Conoptics, Inc., USA), a ELWD 40x/0.6 NA objective with a correction collar for up to 2 mm of glass (Nikon Instruments, Inc., Japan), and Slidebook 6 software (Intelligent Imaging Innovations, Inc., USA) for controlling the microscope and for image acquisition. The correction collar on the objective lens was adjusted to optimize image quality when imaging deeper into tissue, as previously described^3^. A pixel dwell time of 2 µs was used with pixel averaging (5/scan) to acquire three-dimensional (z-stack) images composed of 60–100 images with a 1- or 2-μm step size^18,19^.

Baseline images were acquired three weeks after GRIN lens implantation and one week prior to the midline fluid percussion injury or middle cerebral artery occlusion procedure. Post-injury images were acquired at 1 hour, and 3, 7, 14, 30 and 60 days after the fluid percussion injury and 24 hours following 60-min of MCAo. Image sets were exported and stored as TIFF files on an external hard drive for offline analysis.

### Adapted fluid percussion injury

The midline fluid percussion injury model in mice was conducted as previously described^16^, with an adaptation made during the attachment of the injury hub. The animal was initially anesthetized with 5% isoflurane. Once the animal no longer exhibited a righting reflex, it was transferred to a stereotaxic frame equipped with ear bars and nose cone for delivery of 1–1.5% isoflurane. The surgical site was cleaned with 91% isopropyl and iodine. Once the area was dry, a 1.65-mm diameter by 1-mm long section of plastic trimmer line (Weed Eater®) was glued on the midline between bregma and lambda with liquid cyanoacrylate (Krazy Glue®). The plastic post served as a guide for a trephine to create a craniectomy^66^.

After the cyanoacrylate dried, a 3 mm craniectomy was created with a trephine (3-mm outside diameter, Salvin Dental Specialties, Inc., USA) mounted in a pin vice. Trephining continued until the overlying layer of dental acrylate and bone flap was moved independently of the skull. Care was taken not to cut into the dura with the trephine. If the dura was compromised, the animal was removed from the study. The bone flap was removed by pulling on the piece of string trimmer line. If the trimmer line came off prior to removal, a pair of fine forceps (0.6-mm tip, Roboz Surgical Instrument Company, USA) was used to gently remove the bone flap without piercing the dura. Once the bone flap was removed, cotton pads and sterile 0.9% saline were used to control bleeding.

Injury hubs were constructed using 24 G hypodermic needles. The needle hub was cut just above the second ridged line so that the hub opening could fit over the 3 mm diameter craniectomy^66^. The hub was placed over the craniectomy and secured with gel cyanoacrylate (Loctite®). Next, Kwik-Sil, (World Precision Instruments, LLC) a biocompatible, silicone adhesive was spread around the injury hub in a circular motion, beginning at the base and ending halfway up the injury hub. The seal was approximately 2.5 mm thick and covered the washer portion of the GRIN lens, but not the lens. After allowing 5 minutes for the Kwik-Sil to cure, dental cement (Lang Dental Manufacturing Company, Inc., USA) was applied over the Kwik-Sil and halfway up the side of the injury hub (Fig. 2a) to reinforce the injury hub. Once the dental cement was dry, the hub was filled with 0.9% saline and checked for leaks. Any leaks were sealed before the animal was removed from anesthesia and transferred to a heating pad for recovery. To keep the dura moist, a small piece of tape was placed over the injury hub to retain saline in the hub. Animals were singly housed overnight.

Dental acrylic was applied over the Kwik-Sil, which is an elastomer, to prevent expansion of the elastomer during fluid percussion. Without dental cement covering the Kwik-Sil, this expansion resulted in inconsistent degrees of brain injury to animals in preliminary studies. Once dental cement was applied over the Kwik-Sil, a consistent level of brain injury was achieved. Care was taken to only apply dental cement over the Kwik-Sil and not on the head plate or lens (Fig. 2). If dental acrylic was applied directly to the head plate, the plate was occasionally pulled off during injury hub removal. The injury hub cannot be left in place after fluid percussion injury because the hub does not allow placement of a microscope objective over the GRIN lens. Also, it is best to remove the hub to seal the craniectomy with a glass cover slip to prevent infection.

Animals were randomly divided into either the sham injury (n = 4) or FPI (n = 4) group. Each animal was anesthetized with 5% isoflurane for five minutes in an anesthesia chamber (Kent Scientific Corp., USA). Immediately after removal from the chamber, the injury hub was filled with 0.9% sterile saline and connected to the fluid percussion injury device (Custom Design & Fabrication, Virginia Commonwealth University, Richmond, VA). Anesthesia was withdrawn and when a response to toe pinches was observed, but before the animal had fully regained consciousness, the pendulum was released^66^. After impact, the injury hub was detached from the percussion device and the mouse was placed on a heating pad until it righted itself (Braintree Scientific, Inc., USA). The elapsed time from injury to observation of the righting reflex was recorded. For a moderate injury, a righting response between 6–10 minutes was required. For sham injured animals, the same procedure was followed except, the pendulum was not allowed to hit the fluid-filled cylinder. After the animal had righted itself and the parameters of injury were recorded, the animal was returned to its home cage until it was time to remove the injury hub.

To seal the craniectomy after injury, the animal was briefly re-anesthetized and then transferred to a stereotaxic frame equipped with ear bars and a nose cone. Anesthesia was maintained at 1.5%. The injury hub was carefully removed by gently pulling away the Kwik-Sil with forceps. Next, sterile saline was used to clean the area before sealing. After the area was cleaned, 1.5% low melt agarose (IBI Scientific, Peosta, Iowa) was placed on the craniectomy followed by a #1.5 cover glass, 3 mm in diameter (Electron Microscopy Sciences, Hatfield, Pennsylvania, USA). Once the agarose had solidified, gel control cyanoacrylate (Loctite®) was carefully placed around the edges of the cover glass. Care was taken to not allow the cyanoacrylate to run under the cover glass and agarose and onto the dura mater. Once the cyanoacrylate had cured (approximately five minutes), Aplicap™ dental adhesive (RelyX ™ Unicem, 3M Corp., USA) was placed on the skull, beginning around the edge of the cover glass until it had completely covered any openings to the skull. The adhesive was allowed to cure (~20 min) before transferring the animal to the TRIO system^18^ for immediate imaging.

### Removal of mice from experiments

While all implanted mice were successfully imaged after implantation, a few conditions caused the removal of mice from an experiment. Due to the severity of TBI and MCAo injury models, some mice died (~20% in mFPI and ~30% in MCAo). Additionally, technical failures, such as the failure to meet a minimum neurological severity score after an injury, may require removal of a mouse from the study. We did not remove any mice due to insufficient injury in the work presented here. However, mice were removed due to head plates becoming detached.

### Pre- and post-imaging following middle cerebral artery occlusion (MCAo)

Singlet GRIN lenses (d = 0.5 mm, l = 1.6 mm) were attached to a 5-mm diameter #1.5 cover glass and stainless-steel washer with optically clear adhesive (NOA 71). The end of the lens was implanted into layer 5 of the cortex of Cx3cr1-tdTomato positive mice as previously described (see Methods, hrGRIN lens system implantation). Approximately 3–4 weeks following surgery, baseline images were acquired using a multiphoton microscope. Mice were briefly anesthetized with 5% isoflurane and then transferred to a TRIO platform imaging support system, as previously described^18^. Anesthesia was maintained throughout the imaging session with 1.5% isoflurane. A Chameleon Vision-2 multiphoton laser (80 MHz, Coherent) tuned to 1020 nm was used to excite tdTomato fluorescence. Emitted light was filtered through a 612/69 nm bandpass filter (Brightline^®^ filters, Semrock, Inc). Scanned images were acquired on the same microscope system described in the Longitudinal Imaging section. Most neuronal cell processes are not completely captured within one image plane; thus, we acquire multiple images at increasing depths (z-stacks). A pixel dwell time of 2 µs was used with pixel averaging (5/scan) to acquire three-dimensional (z-stack) images composed of 61 images with a 1 μm step size. Approximately one week following baseline imaging, mice underwent sham or transient (60 minute) MCAo induced by the occlusion of the right middle cerebral artery using an intraluminal filament method, as previously described^17^. Post-I/R images were acquired 20–24 hours following MCAo. Image sets were exported and stored as TIFF files on an external hard drive for offline analysis.

### Vascular counterstaining

Texas red dye conjugated to 70 kDa dextran or FITC dye conjugated to 70 kDa dextran (Life Technologies Corporation, USA) was injected via the tail vein. Powdered dyes were prepared with 0.9% sterile saline (0.1 mg/mL). Animals were briefly anesthetized with 5% isoflurane and transferred to an acrylic restraint device (Kent Scientific Corporation, USA). Tails were cleaned with 91% isopropyl and prepared dye solution (100 μl per animal) was injected, using a 30-ga sterile hypodermic needle, approximately five minutes prior to image sessions. Dyes were visible for the entire imaging session (~1 hr) and no additional injections were necessary.

### Image analysis

Exported 16-bit TIFF files were analyzed using ImageJ (v1.51i) software (NIH). Each stack for each time point was realigned using “StackReg”, a downloadable plug-in, to correct minor misalignment of successive images caused by motion artifacts^67^, such as brain movement from blood flow. Large motion artifacts are prevented with secure head fixation, but this does not prevent micromotion. Micromotion can sometimes occur while an image plane is being acquired. In this case only part of the image can be aligned which results in some blurring in one part of the image. After correcting image alignment, an average intensity z-projection of several adjacent image panes was created for each time point. An identical field of view was chosen for each time point to track the same axons over time. To compare microglia activation before and after MCAo, imaging planes were randomly chosen between the middle and the end of the z-stack and an average intensity z-projection was created for each time point.

### Quantification of damage following mFPI

At each time-point the number of axons with undulations, varicosities, or terminal bulbs was measured by an individual blinded to injury condition.

#### Undulations

Axons were counted as undulated if they had a rippled profile as compared to their baseline image. These were counted as undulated until they either recovered to their baseline morphology or the axons developed varicosities or a terminal bulb.

#### Varicosities

Axons with varicosities were counted if swellings were at least 1.5x the diameter of the axon immediately proximal and/or distal to the swelling^22,25^. When axons intersect one another, the fluorescence intensity becomes brighter in that area. As a result, intersections can be confused as swellings. In order to determine if bright areas on axons were intersections or varicosities, the observer examined individual planes in the image z-stack. Only swellings without axonal intersections were counted as varicosities.

#### Terminal bulbs

Terminal bulbs were defined as the distal end of disconnected axons with a large swelling that was at least 2X the diameter of the axon. Individual planes in the image z-stack were inspected to confirm that the axon was not connected in another plane before counting it as a terminal bulb.

#### Percentage of damage at each time point

The total number of axons with each feature (F_A_) was divided by the total number of axons (T_A_) in the field of view at each time-point to obtain the percentage of damaged axons (F_A_/T_A_ * 100) (Fig. 4). In order to compare new damage at each time-point (Fig. 5), only newly developed features (i.e., not present in previous time-points) were measured. The total number of axons with new damage (N_A_), including new axons with varicosities, undulated axons progressing to axons with varicosities, axons with varicosities progressing to terminal bulbs, and terminal bulbs resulting in axon loss) was divided by the total number of axons (T_A_) in the field of view to obtain a percentage of newly damaged axons at each time-point (N_A_/T_A_ *100).

### Statistical analysis

Two-way ANOVA with Sidak correction was used to obtain statistical significance unless otherwise noted. Statistical significance was defined as p < 0.05. Sample sizes and p-values are noted in the figure legends. Data are presented as mean ± SEM.

### 3D print methods and data sharing

All 3-dimensional print files for the TRIO mFPI Head Plate (Figs 2 and 10) and for the TRIO Imaging Support Platform^18^ are available at the National Institutes of Health 3D Print Exchange at https://3dprint.nih.gov/discover/3dpx-010720, Model ID 3DPX-010720. Images and metadata are available upon request to Dr. Teresa Murray, tmurray@latech.edu.

## Supplementary information

Supplementary Information

Video S1
