# Supplementary material for: Time course images of cellular injury and recovery in murine brain with high-resolution GRIN lens system
Source: Sci Rep. 2019 May 28;9:7946. doi: 10.1038/s41598-019-44174-7 (PMC6538613; doi:10.1038/s41598-019-44174-7)
Supplement: Supplementary file 1 — Supplementary Information [file 41598_2019_44174_MOESM1_ESM.pdf]

Supplementary Information

Time course images of cellular injury and recovery in murine brain with high-resolution GRIN lens system

Chelsea D. Pernici[1], Benjamin S. Kemp[1], Teresa A. Murray[1,*]

[1] Center for Biomedical Engineering and Rehabilitation Sciences, Louisiana Tech University, Ruston, LA, USA

* Corresponding author: Teresa A. Murray, PhD, tmurray@latech.edu,
Alternate e-mail, bioengineer1@hotmail.com

Supplemental Data. ***Video S1.*** An in vivo, time-lapse video of the cortex with a blood vessel was acquired in a Cx3cr1-tdTomato mouse after MCAo. Microglia can be observed moving through the tissue surrounding the blood vessel. Additionally, monocytes and neutrophils can be observed flowing through the blood vessel. The capability of imaging microglia/macrophage dynamics would be useful in studies that assess infiltration and the resolution of inflammation. Scale bar = 10 µm.
